# Homogenization modelling of antibiotic diffusion and adsorption in viral liquid crystals

**DOI:** 10.1098/rsos.221120

**Published:** 2023-01-04

**Authors:** M. T. van Rossem, S. Wilks, P. R. Secor, M. Kaczmarek, G. D’Alessandro

**Affiliations:** ^1^ Physics and Astronomy, University of Southampton, Southampton SO17 1BJ, UK; ^2^ Health Sciences, University of Southampton, Southampton SO17 1BJ, UK; ^3^ Mathematical Sciences, University of Southampton, Southampton SO17 1BJ, UK; ^4^ Division of Biological Sciences, University of Montana, Missoula, MT 59812, USA

**Keywords:** modelling, liquid crystals, biofilms, *P. aeruginosa*, homogenization, diffusion

## Abstract

Systems of rod-shaped viruses have long been important to the science of living liquid crystals, as their monodispersity and uniform charge make them convenient model systems. Recently, it was shown that, upon the addition of polymers, suspensions of rod-shaped viruses form liquid crystals that are linked with increased tolerance of bacteria against antibiotics. We use homogenization to obtain effective equations describing antibiotic diffusion through these liquid crystals. The analytical results of homogenization are compared with numerical results from an exact microscopic model, showing good agreement and thus allowing us to identify the key parameters behind the process. Our modelling shows that the adsorption plays a key role in increasing antibiotic diffusion time and therefore the presence of nematic rod-shaped viruses may increase antibiotic tolerance through physical mechanisms alone. These results demonstrate the applicability of homogenization as an analytical tool to systems of liquid crystalline viruses, with relatively straightforward extension to more complex problems such as liquid crystalline biofilms, other biological liquid crystals and biological systems with different types of local structural order.

## 1. Introduction

Liquid crystals are ubiquitous in biology; an early example is Reinitzer’s discovery of liquid crystals in experiments on carrot extract [1]. Furthermore, throughout the rich history of the topic, mathematical models of liquid crystals have frequently been inspired by, and applied to, suspensions of rod-shaped viruses due to their high degree of uniformity [2], starting with the celebrated Onsager theory of phase transitions in lyotropic liquid crystals [3]. Recently, the topic of liquid crystalline viral suspensions has taken a new turn, with the discovery that the filamentous virus Pf4 forms liquid crystals in *Pseudomonas aeruginosa* biofilms [4], which are bacterial communities with a complex structure; the bacteria are surrounded by a self-produced extracellular matrix consisting of polymer substances.

Pf4 is a bacteriophage (henceforth phage): a virus that infects and undergoes replication within bacteria. It has negative surface charge and is approximately 2 μm long and 6–7 nm in diameter. In contrast to many other types of phages, Pf4 virions do not require host cell lysis to replicate, developing a mutualistic relationship with their host. For Pf4, the host bacteria are *P. aeruginosa*, with Pf4 infecting cells and also replicating within. Hence, the liquid crystal formation occurs naturally in biofilms of *P. aeruginosa*, where they lead to increased tolerance against antibiotics [4–6].

Antibiotic tolerance is the transient ability of bacteria to withstand a certain duration of antibiotic treatment, for instance, through arresting bacterial growth. This is not to be confused with antibiotic resistance, which is the inherent ability of bacteria to withstand a certain concentration of antibiotics, regardless of treatment duration, and is vertically transmitted on to future generations, leading to resistant populations (i.e. always due to genetic mutations). Antibiotic tolerance and resistance can be induced by exposure to sub-lethal concentrations of antibiotics. The ability of bacteria to withstand antibiotics is a growing area of concern in medicine and human health, and an important area of research. *In vitro*, upon mixing with polymers that occur naturally in *P. aeruginosa* biofilms, the filamentous virus Pf4 forms liquid crystalline droplets called tactoids [4]. An increased antibiotic tolerance has been observed in bacteria encapsulated by Pf4 tactoids [6]. This may be due to a purely physical barrier effect or the ability of phages, being anionic, to adsorb cationic antibiotics [4,7]. We use homogenization to study the relative importance of these two factors and assess their relevance to antibiotic tolerance.

Most of the polymers in *P. aeruginosa* biofilms are negatively charged, thus not interacting with the anionic phages or each other, in the absence of multivalent cations that induce crosslinking [4]. This means that the polymers can act as depleting agents according to the theory of Asakura & Oosawa [8]. Furthermore, phase behaviour of rod-shaped components of biofilms, such as Pf4 virions, can be described by Onsager’s theory [3]. Formation of liquid crystals has also been studied for other biofilms than *P. aeruginosa* [9,10]. However, the topic is relatively novel and mathematical modelling of liquid crystalline biofilms has been limited to cell-level simulations [9,10] and population dynamics [11].

In this paper, we propose an analytically solvable continuum model to describe the diffusion and adsorption antibiotics in liquid crystals formed by Pf4 virions. To achieve an analytical solution for the complex microscopic structure of a liquid crystal, we apply the method of homogenization. This mathematical technique describes systems in which scale separation occurs, so that the physics at a macroscopic scale, and a much smaller microscopic scale, can be disentangled [12]. If the details of such a system are regular on a microscopic scale, they can be homogenized; this means that the microscopic structure is averaged out and what is left is a much simpler macroscopic structure, governed by effective equations. These give the same results as the microscopic model, in the limit that the microscopic scale becomes infinitesimal. The applicability of homogenization and the form of the effective equations depend on the scaling of the parameters, hence each scaling regime needs to be considered separately. An overview of the scaling regime dependence of homogenization for diffusion and reaction in porous media has been given by Battiato & Tartakovsky [13].

Homogenization is applicable to liquid crystalline structures, since these are orientationally ordered on a microscopic scale; the microscopic structure can be considered as a lattice of aligned phages and we can straightforwardly define a small length scale of a lattice unit cell. For instance, homogenization has been applied to liquid crystals by Bennett *et al.*, among others [14–17]. In the homogenization of diffusion and adsorption in liquid crystals, we exploit the similarity with reactive diffusion in porous media, since homogenization has been widely applied in mathematical descriptions of this phenomenon [13,18–24]. Hence, the homogenization of the model in this paper follows the approach of Allaire [19].

We obtain a macroscopic, homogenized model, which can be compared in some simpler cases with an exact, microscopic model solved using Comsol. These models serve to verify that homogenization is a valuable means of analysis in the topic of liquid-crystal-induced antibiotic tolerance and biological liquid crystals in general. We demonstrate this by showing that the homogenized model offers clear insight into the parameters governing the system, allowing for efficient analysis. Furthermore, the model is straightforwardly extendable to more complex geometries. Because our research is interdisciplinary, it has two aspects: one physical and mathematical, and one biological. As a consequence, we also obtain a set of biologically oriented results, concerned with whether tactoids can cause antibiotic tolerance. These results are presented elsewhere in full detail [25].

The structure of this paper is as follows. In §2, we discuss and non-dimensionalize the microscopic model and restrict it to two dimensions; its homogenized form is derived in §3, where we also test its agreement with the microscopic model. The homogenized model is extended to three dimensions in §4. The results are presented and discussed in §5, where we discuss two- and three-dimensional phage configurations. A brief summary of the results and future outlook concludes this work.

## 2. The microscopic model

**Figure 1.**
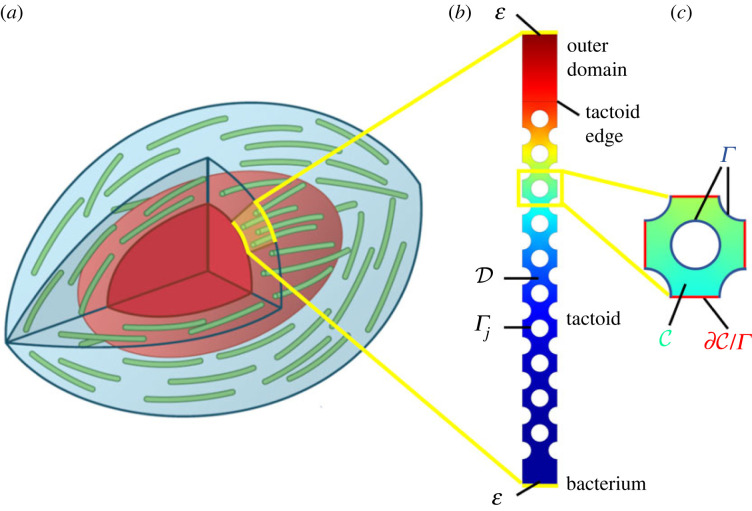
A schematic illustration of the model: (*a*) a sketch of a tactoid (in blue), with phages (green) surrounding a bacterium (red). Shaded in yellow is an example of a two-dimensional tactoid slice on which the model is solved. This domain is shown in (*b*). The colour scale indicates a typical antibiotic concentration, from high (red) to low (blue), and circles represent phages. A unit cell of this domain is enlarged in (*c*), with the indices *j* dropped for notational convenience.

We solve the problem of antibiotic diffusion in a three-dimensional liquid crystal and consider a few of its applications. The first of these is antibiotic diffusion in a tactoid consisting of orientationally ordered phages, encapsulating a bacterium, as illustrated in figure 1. Throughout this paper, we assume that the phage liquid crystalline state has reached equilibrium, which means that the antibiotics are added to the system after the nematic state has formed. Images by Tarafder *et al.* [6] show that such a phage liquid crystalline droplet encapsulating a bacterium has planar anchoring at its boundaries and that the nematic director follows the curvature of the tactoid. However, curvature effects can be neglected because the large aspect ratio of the phages ensures that the geometry is locally flat and hence the phage alignment is locally parallel. Due to the nematic order, one can consider the internal tactoid structure to consist of filaments that form a regular lattice. This is not a restrictive hypothesis, since the results obtained are very robust to fluctuations on the microscopic scale. This has been proved by Bruna & Chapman for diffusion in porous media [26] and verified for our results in appendix S1 in the electronic supplementary material. The tactoid geometry allows for further simplifications. As the phages are long and thin (see appendix S2 in the electronic supplementary material) and aligned, diffusion along the length of the phages will be insignificant compared with diffusion across the phages. Therefore, the system can be reduced to two dimensions. Due to the regularity of the lattice and the insignificance of curvature effects, we can choose a tactoid sector with a thickness of one lattice unit cell as a domain. Under these reasonable assumptions, the two-dimensional domain is representative of a tactoid in its entirety when the phage packing density is constant across the tactoid. This last assumption has little influence on the antibiotics diffusion, as discussed in §5. The resulting domain is shown in figure 1. Its height covers the entire thickness of the tactoid, from the bacterium boundary to the outer tactoid edge. It is one phage sector thick and the vertical outer boundary conditions in figure 1 are periodic.

In its dimensional form, the microscopic model to be homogenized is expressed as

2.1a $$\frac{\partial \tilde{u}}{\partial \tilde{t}}=\tilde{\nabla }\cdot (\tilde{D}\tilde{\nabla }\tilde{u}),\;\tilde{x}\in \tilde{D},$$

2.1b $$\frac{\partial \tilde{v}}{\partial \tilde{t}}=\tilde{\kappa }(\tilde{\alpha }\tilde{u}-\tilde{v}),\;\tilde{x}\in {\tilde{\Gamma }}_{\;j},$$

2.1c $$-\tilde{D}n\cdot \tilde{\nabla }\tilde{u}=\tilde{\kappa }(\tilde{\alpha }\tilde{u}-\tilde{v}),\;\tilde{x}\in {\tilde{\Gamma }}_{\;j}$$

2.1d $$\mathrm{and}\;\;n\cdot \tilde{\nabla }\tilde{u}=0,\;\tilde{x}\in \tilde{E},$$

where $\tilde{u}$ denotes the volume concentration of free antibiotics (in units of m^−3^), $\tilde{v}$ is the surface concentration of adsorbed antibiotics (in units of m^−2^), $\tilde{D}$ is the diffusion coefficient in m^2^/s, $\tilde{\alpha }$ is the equilibrium binding coefficient in m, $\tilde{\kappa }$ is the adsorption rate in 1/s and ***n*** is the outward unit normal pointing into the phages. Equation (2.1*a*) describes the diffusion in the tactoid domain $\tilde{D}$ and equation (2.1*b*) describes the adsorption at the phage boundary ${\tilde{\Gamma }}_{\;j}$ (where the index *j* denotes the *j*th phage), determining $\tilde{v}$. From this, equation (2.1*c*) determines the flux at the phage boundary and equation (2.1*d*) is a no-flux boundary condition at the domain end boundaries $\tilde{E}$, which is the union of the top boundary in figure 1 and the bacterium boundary. Equation (2.1*d*) can be replaced with other conditions if necessary, without invalidating the analysis presented here. The adsorption dynamics in equation (2.1*b*) are linear, which is justified since the degree of adsorption is low compared with the phage adsorption capacity, as shown from the parameter estimates in appendix S2 of the electronic supplementary material.

We will proceed to write the microscopic model in non-dimensional form, which will allow us to identify the key control parameters for the dynamics and also to homogenize the model. There are two length scales in this problem: the tactoid thickness $\tilde{L}$ and the interphage distance scale $\tilde{a}$. We define non-dimensional macroscopic spatial coordinates by scaling with $\tilde{L}$

2.2 $$x=\frac{\tilde{x}}{\tilde{L}}.$$

The two spatial scales naturally engender two separate diffusion timescales. The first is the diffusion time at the microscopic (phage) scale,

2.3 $${\tilde{\tau }}_{D}=\frac{{\tilde{a}}^{2}}{\tilde{D}},$$

while the second is the diffusion time at the macroscopic (tactoid) scale, which is much larger,

2.4 $${\tilde{\tau }}_{D}^{\left(M\right)}\equiv \frac{{\tilde{L}}^{2}}{\tilde{D}}=\frac{{\tilde{\tau }}_{D}}{\epsilon^{2}}.$$

As $\tilde{a}\ll \tilde{L}$, we define a small parameter ϵ as $\epsilon \equiv \tilde{a}/\tilde{L}=N^{-1}\ll 1$, where *N* is the number of phage layers across the tactoid. This number quantifies how many phages can fit across the thickness of a tactoid; in other words, the difference in scale between phage and tactoid. Another aspect of the antibiotic dynamics in the tactoid is the adsorption at the phage surface. This happens on a timescale of

2.5 $${\tilde{\tau }}_{\kappa }=\frac{1}{\tilde{\kappa }},$$

which is assumed to be of the same order of magnitude as ${\tilde{\tau }}_{D}$, as discussed in appendix S2 in the electronic supplementary material. This is one of the scaling choices for which microscopic and macroscopic scales are separated and hence homogenization is applicable. Furthermore, it implies that adsorption is fast compared with macroscopic diffusion, which is realistic. We scale the time coordinate with the macroscopic diffusion time as follows:

2.6 $$t=\frac{\tilde{t}}{{\tilde{\tau }}_{D}^{\left(M\right)}}=\frac{\tilde{t}\tilde{D}}{{\tilde{L}}^{2}}.$$

Finally, the antibiotic concentrations are scaled as

2.7a $$\tilde{u}={\tilde{u}}_{0}u$$

and

2.7b $$\tilde{v}={\tilde{v}}_{0}v.$$

The scaling parameters ${\tilde{u}}_{0}$ and ${\tilde{v}}_{0}$ will be set later on. Substituting equations (2.2)–(2.7) leads to the following form of equations (2.1):

2.8a $$\frac{\partial u}{\partial t}=\nabla^{2}u,\;x\in D,$$

2.8b $$\frac{\partial v}{\partial t}=\frac{\tilde{\kappa }{\tilde{L}}^{2}}{\tilde{D}}\left(\frac{\tilde{\alpha }{\tilde{u}}_{0}}{{\tilde{v}}_{0}}u-v\right),\;x\in \Gamma_{\;j},$$

2.8c $$-n\cdot \nabla u=\frac{\tilde{\kappa }\tilde{L}{\tilde{v}}_{0}}{\tilde{D}{\tilde{u}}_{0}}\left(\frac{\tilde{\alpha }{\tilde{u}}_{0}}{{\tilde{v}}_{0}}u-v\right),\;x\in \Gamma_{\;j}$$

2.8d andn⋅∇u=0,x∈E,

where D and $\Gamma_{\;j}$ are the tactoid domain and *j*th phage boundary in non-dimensional units of ***x***, respectively, and E is the union of non-dimensional domain end boundaries. We define

2.9 $$\gamma =\frac{\tilde{\kappa }{\tilde{L}}^{2}}{\tilde{D}}∼O\left(\frac{1}{\epsilon^{2}}\right)$$

and

2.10 $$\mu =\frac{\tilde{\alpha }{\tilde{u}}_{0}}{{\tilde{v}}_{0}}.$$

Some comments are in order about the scale of *μ*, which will be used to set the parameters ${\tilde{u}}_{0}$ and ${\tilde{v}}_{0}$. We define the free and bound antibiotic concentrations at equilibrium as *u*_∞_ and *v*_∞_, and observe from equation (2.8*b*) that

2.11 $$v_{\infty }=\frac{\tilde{\alpha }{\tilde{u}}_{0}}{{\tilde{v}}_{0}}u_{\infty }.$$

The total amount of bound antibiotics at equilibrium is

2.12 $${\tilde{v}}_{\mathrm{Tot}}\propto N^{2}\tilde{a}{\tilde{v}}_{0}v_{\infty },$$

since the total number of phages scales as *N*^2^ and the phage size is of the order $\tilde{a}$. Combining equations (2.11) and (2.12) gives

2.13 $${\tilde{v}}_{\mathrm{Tot}}\propto \frac{1}{\epsilon^{2}}\tilde{a}{\tilde{v}}_{0}\frac{\tilde{\alpha }{\tilde{u}}_{0}}{{\tilde{v}}_{0}}u_{\infty }=\frac{\tilde{L}\tilde{\alpha }{\tilde{u}}_{0}}{\epsilon }u_{\infty }.$$

This means that ${\tilde{v}}_{\mathrm{Tot}}$ diverges as ϵ→0, or equivalently *N* → ∞ unless

2.14 $$\tilde{\alpha }=\epsilon {\tilde{\alpha }}_{1}\;\mathrm{and}\;\alpha_{1}≃O(1).$$

Now, we can set the ratio of ${\tilde{u}}_{0}$ and ${\tilde{v}}_{0}$ by requiring that the total amounts of free and bound antibiotics are of the same order,

2.15 $${\tilde{L}}^{2}{\tilde{u}}_{0}∼N^{2}\tilde{a}{\tilde{v}}_{0}=\frac{1}{\epsilon^{2}}\epsilon \tilde{L}{\tilde{v}}_{0}\;⟹\;{\tilde{u}}_{0}=\frac{{\tilde{v}}_{0}}{\tilde{a}}.$$

We cannot fix ${\tilde{u}}_{0}$ and ${\tilde{v}}_{0}$ individually unless one of the outer boundary conditions is Dirichlet, since the system is scale invariant. The scaling in equation (2.15) also determines the scaling of *μ*,

2.16 $$\mu =\frac{\tilde{\alpha }{\tilde{u}}_{0}}{{\tilde{v}}_{0}}=\frac{\tilde{\alpha }}{\tilde{a}}=\frac{\epsilon {\tilde{\alpha }}_{1}}{\tilde{a}}=\frac{{\tilde{\alpha }}_{1}}{\tilde{L}}=O(1).$$

Finally, we can substitute equations (2.9), (2.15) and (2.10) into equations (2.8), which results in the non-dimensional microscopic model

2.17a $$\frac{\partial u}{\partial t}=\nabla^{2}u,\;x\in D,$$

2.17b $$\frac{\partial v}{\partial t}=\gamma (\mu u-v),\;x\in \Gamma_{\;j},$$

2.17c $$-n\cdot \nabla u=\epsilon \gamma (\mu u-v),\;x\in \Gamma_{\;j}$$

2.17d andn⋅∇u=0,x∈E.

We apply homogenization to this model in the next section and solve it numerically in §4.

## 3. The two-dimensional homogenized model

Having defined the non-dimensional microscopic model in equations (2.17), we can obtain the effective macroscopic equations using homogenization. This is useful since, due to the microscopic structure, the model cannot be solved analytically and numerical modelling is computationally expensive. Homogenization can be applied due to the nematic order; we can consider the microscopic structure formed by the phages to be a lattice of locally periodic unit cells. Homogenizing the model results in effective equations on a simple macroscopic geometry consisting of a filled rectangle, i.e. without phage structure.

The ratio between the microscopic unit cell size and the macroscopic tactoid width is given by the small parameter ϵ. To homogenize, we introduce a microscopic coordinate ***y*** defined in a unit cell (figure 1) and expand the fields in powers of ϵ, with

3.1a $$y=\frac{x}{\epsilon },$$

3.1b $$u=\epsilon^{k}u_{k}(x,y,t)$$

3.1c $$\mathrm{and}\;\;\;v=\epsilon^{k}v_{k}(x,y,t),$$

where summation over repeated indices is implied. Substitution into equations (2.17) gives

3.2a $$\epsilon^{k}\frac{\partial u_{k}}{\partial t}=\left(\frac{1}{\epsilon^{2}}\nabla_{y}^{2}+\frac{2}{\epsilon }\nabla_{x}\cdot \nabla_{y}+\nabla_{x}^{2}\right)\epsilon^{k}u_{k},\;y\in C,$$

3.2b $$\epsilon^{k}\frac{\partial v_{k}}{\partial t}=\frac{\gamma_{2}}{\epsilon^{2}}(\mu u_{k}-v_{k})\epsilon^{k},\;y\in \Gamma$$

3.2c $$\mathrm{and}\;\;-n\cdot \left(\frac{1}{\epsilon }\nabla_{y}+\nabla_{x}\right)u_{k}\epsilon^{k}=\frac{\gamma_{2}}{\epsilon }(\mu u_{k}-v_{k})\epsilon^{k},\;y\in \Gamma ,$$

where $\gamma_{2}=\gamma \epsilon^{2}=O(1)$, as required by equation (2.9), and C and Γ are the non-dimensional free unit cell domain and phage boundary, respectively, as shown in figure 1. Now, we can expand equations (3.2*a*) to (3.2*c*) in ϵ and analyse them order by order.

### 3.1. Leading order

At this order, the equations are

3.3a $$\nabla_{y}^{2}u_{0}=0,\;y\in C,$$

3.3b $$\gamma_{2}(\mu u_{0}-v_{0})=0,\;y\in \Gamma$$

3.3c $$\mathrm{and}\;\;n\cdot \nabla_{y}u_{0}=\gamma_{2}(\mu u_{0}-v_{0}),\;y\in \Gamma .$$

These equations are solvable and have the solutions

3.4a $$u_{0}(x,y,t)=u_{0}(x,t)$$

and

3.4b $$v_{0}(x,t)=\mu u_{0}(x,t).$$

### 3.2. First order

At next order in ϵ, the equations are

3.5a $$\nabla_{y}^{2}u_{1}+2\nabla_{x}\cdot \nabla_{y}u_{0}=0,\;y\in C,$$

3.5b $$\gamma_{2}(\mu u_{1}-v_{1})=0,\;y\in \Gamma$$

3.5c $$\mathrm{and}\;\;\;-n\cdot (\nabla_{y}u_{1}+\nabla_{x}u_{0})=\gamma_{2}(\mu u_{1}-v_{1}),\;y\in \Gamma .$$

The solution of equation (3.5*b*) is

3.6 $$v_{1}(x,y,t)=\mu u_{1}(x,y,t).$$

Equations (3.5*a*) and (3.5*c*) are solvable. Their solution has the form

3.7 $$u_{1}(x,y,t)=\chi (y)\cdot \nabla_{x}u_{0}(x,t),$$

where the components *χ*_*k*_ of the (vector) function ***χ***(***y***) are the solution of the cell problem

3.8a $$\nabla_{y}^{2}\chi_{k}=0,\;y\in C$$

and

3.8b $$-n\cdot \nabla_{y}\chi_{k}=n_{k},\;y\in \Gamma ,$$

with *n*_*k*_ is the *k*th component of the outward unit normal from the unit cell C into the phages. This equation generally needs to be solved numerically but only once, since it yields a generally applicable effective diffusion coefficient for the microscopic geometry.

### 3.3. Second order

At this order in the smallness parameter, the equations are

3.9a $$\frac{\partial u_{0}}{\partial t}=(\nabla_{y}^{2}u_{2}+2\nabla_{x}\cdot \nabla_{y}u_{1}+\nabla_{x}^{2}u_{0}),\;y\in C,$$

3.9b $$\frac{\partial v_{0}}{\partial t}=\gamma_{2}(\mu u_{2}-v_{2}),\;y\in \Gamma$$

3.9c $$\mathrm{and}\;\;-n\cdot (\nabla_{y}u_{2}+\nabla_{x}u_{1})=\gamma_{2}(\mu u_{2}-v_{2}),\;y\in \Gamma .$$

The solvability condition is obtained by integrating equation (3.9*a*) over the unit cell C, and applying the divergence theorem and the boundary condition in equation (3.9*c*) as follows:

3.10 $$\begin{array}{rl} 0 & =\int_{C}-\frac{\partial u_{0}}{\partial t}+[\nabla_{y}\cdot (\nabla_{y}u_{2}+\nabla_{x}u_{1})+\nabla_{x}\cdot (\nabla_{y}u_{1}+\nabla_{x}u_{0})]\;d^{3}y\\ & =\int_{\Gamma }n\cdot (\nabla_{y}u_{2}+\nabla_{x}u_{1})\;d^{2}y-\int_{C}\frac{\partial u_{0}}{\partial t}\;d^{3}y+\int_{C}\nabla_{x}\cdot (\nabla_{y}u_{1}+\nabla_{x}u_{0})\;d^{3}y\\ & =-\int_{\Gamma }\frac{\partial v_{0}}{\partial t}\;d^{2}y-\int_{C}\frac{\partial u_{0}}{\partial t}\;d^{3}y+\int_{C}\nabla_{x}\cdot (\nabla_{y}u_{1}+\nabla_{x}u_{0})\;d^{3}y\\ & =-|\Gamma |\frac{\partial v_{0}}{\partial t}-|C|\frac{\partial u_{0}}{\partial t}+|C|\nabla_{x}^{2}u_{0}+\nabla_{x}\cdot \int_{C}\nabla_{y}u_{1}\;d^{3}y. \end{array}$$

Substituting equations (3.4*b*) and (3.7) into this expression, we obtain the homogenized equation

3.11 $$(|C|+\mu |\Gamma |)\frac{\partial u_{0}(x,t)}{\partial t}=\nabla_{x}\cdot [D^{\left(\mathrm{eff}\right)}\nabla_{x}u_{0}(x,t)],\;x\in D_{H},$$

where $D_{H}$ is the non-dimensional homogenized domain that does not contain the microscopic phage structure, and *D*^(eff)^ is the effective diffusion tensor

3.12 $$D_{ij}^{\left(\mathrm{eff}\right)}=|C|+\int_{C}\partial_{y_{i}}\chi_{\;j}(y)\;d^{3}y.$$

The effective diffusion tensor, being derived from the unit cell problem, contains the effect of the microscopic geometry on the diffusion; in this case, the effect of the phages as a physical diffusion barrier. The factor in front of the time derivative contains the contribution of the adsorbed antibiotics, and hence gives the effect of adsorption on the diffusion. We can rewrite the homogenized equation (3.11) in dimensional form, using the time and space scalings given by equations (2.6) and (2.2), respectively:

3.13 $$(|C|+\mu |\Gamma |)\frac{\partial \tilde{u}(\tilde{x},\tilde{t})}{\partial \tilde{t}}=\tilde{\nabla }\cdot ({\tilde{D}}^{\left(\mathrm{eff}\right)}\tilde{\nabla })\tilde{u}(\tilde{x},\tilde{t}),\;\tilde{x}\in {\tilde{D}}_{H},$$

where ${\tilde{D}}_{H}$ is the dimensional homogenized domain and the dimensional diffusion tensor is

3.14 $${\tilde{D}}^{\left(\mathrm{eff}\right)}=\tilde{D}D^{\left(\mathrm{eff}\right)}.$$

One can also express the coefficient of the time derivative in terms of dimensional variables by observing that both Γ and C are measured in microscopic units. Therefore, their dimensional versions are, using the scaling in equation (3.1*a*),

3.15 $$|\tilde{\Gamma }|=\tilde{a}|\Gamma |\;\text{and}\;|\tilde{C}|={\tilde{a}}^{2}|C|.$$

Substituting these two expressions and equation (2.10) into equation (3.13), without changing the right-hand side of the equation to preserve the standard diffusion equation form, yields the dimensional homogenized equation

3.16 $$\frac{1}{{\tilde{a}}^{2}}(|\tilde{C}|+\tilde{\alpha }|\tilde{\Gamma |})\frac{\partial \tilde{u}(\tilde{x},\tilde{t})}{\partial \tilde{t}}=\tilde{\nabla }\cdot ({\tilde{D}}^{\left(\mathrm{eff}\right)}\tilde{\nabla })\tilde{u}(\tilde{x},\tilde{t}),\;\tilde{x}\in {\tilde{D}}_{H}.$$

A remark about the scaling is in order here. The coefficient of the time derivative diverges as the layer number *N* → ∞ unless $\tilde{\alpha }=O(1/N)=O(\epsilon )$; this is indeed the scaling of *α* demanded by the condition *μ* ∼ *O* (1).

Both the microscopic and homogenized model, as given by equations (2.1) and (3.16), respectively, were solved in Comsol. The first was solved on the domain in figure 1*b*. The second was solved on a filled rectangle with the same size: the tactoid and outer layer thicknesses are 1 *μ*m and 0.2 *μ*m, respectively. The general form PDE interface was used for all equations. A Dirichlet boundary condition $\tilde{u}=1$ was set at the edge of the outer layer (the top boundary in figure 1).

**Figure 2.**
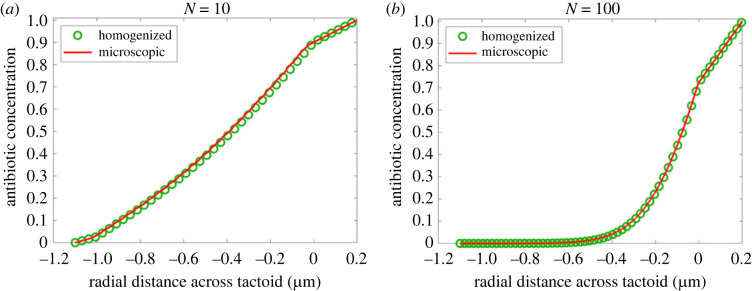
A comparison of antibiotic concentration as a function of the radial tactoid coordinate for the microscopic and homogenized model, at *t* = 0.5 s, for (*a*) 10 phage layers and (*b*) 100 phage layers. The adsorption coefficient *α* = 0.4 μm.

A comparison of the models is presented in figure 2, which shows, for each model, the free antibiotic concentration across the width of a tactoid. The concentration is averaged across the domain width (horizontal in figure 1), resulting in a one-dimensional graph. In the homogenized model, there is no lateral concentration gradient, since the phage structure is averaged out, and the problem can be reduced to one dimension without averaging. Since the small parameter ϵ is determined by the number of phage layers *N*, the agreement is expected to improve with larger *N*. Results are shown for *N* = 10, chosen as a lower limit for the applicability of homogenization since the difference between microscopic and macroscopic scales is merely one order of magnitude, and *N* = 100, a value representative for a real tactoid. The agreement depends on *N* as expected: for *N* = 10, the models agree quite closely; for *N* = 100, the agreement is excellent. This confirms that homogenization is applicable to the present problem.

## 4. The three-dimensional homogenized model

We extend the homogenized model to three dimensions to show further applications. Firstly, this allows us to model a tactoid with varying phage packing density and to assess the influence of this varying density on antibiotic diffusion. As a second example, this model can be applied to a layer of Pf4 liquid crystal of varying thickness with embedded bacteria. The diffusion coefficient is now anisotropic; across the circular phage axis it is still given by ${\tilde{D}}_{\mathrm{eff}}$ and along the long phage axis we take diffusion to be the uninhibited, with diffusion coefficient $\tilde{D}$. However, the homogenized model also contains an adsorption coefficient in front of the time derivative (see equation (3.16)). This coefficient turns out to be equal along both directions of diffusion, which we proceed to show.

The adsorption along the longitudinal direction with respect to the phages was derived using an expansion in the small parameter *η*, which is the difference in scale between the unit cell shown in figure 1 and the phage length ${\tilde{L}}_{\;ph}$: $\eta =\tilde{a}/{\tilde{L}}_{\;ph}$. We are considering diffusion along a three-dimensional channel with the length of a phage and the cross-section corresponding to a unit cell. The space and time variables are scaled as follows:

4.1 $$x_{1}=\frac{{\tilde{x}}_{1}}{{\tilde{L}}_{\;ph}},\;x_{2}=\frac{{\tilde{x}}_{2}}{\tilde{a}},\;x_{3}=\frac{{\tilde{x}}_{3}}{\tilde{a}}\;\mathrm{and}\;t=\frac{\tilde{t}D}{{\tilde{L}}_{\;ph}},$$

where the index 1 indicates the direction along the phages, and 2 and 3 are the directions along the unit cell. On the fast timescale, which can be defined as *t*_2_ = *t*/*η*^2^, we assume that the system has equilibrated. Therefore, the equations do not depend on this timescale. We non-dimensionalize $\tilde{u}$ and $\tilde{v}$ in the same way as in equations (2.7). The diffusion–adsorption equations become

4.2a $$\frac{\partial u}{\partial t}=\frac{\partial^{2}u}{\partial x_{1}^{2}}+\frac{1}{\eta^{2}}\frac{\partial^{2}u}{\partial x_{2}^{2}}+\frac{1}{\eta^{2}}\frac{\partial^{2}u}{\partial x_{3}^{2}},\;x\in D_{3D},$$

4.2b $$-n_{1}\frac{\partial u}{\partial x_{1}}=0,\;x\in \Gamma_{3D},$$

4.2c $$-n_{2}\frac{\partial u}{\partial x_{2}}-n_{3}\frac{\partial u}{\partial x_{3}}=\gamma_{\;ph}(\mu u-v),\;x\in \Gamma_{3D}$$

4.2d $$\mathrm{and}\;\;\;\frac{\partial v}{\partial t}=\frac{\gamma_{\;ph}}{\eta^{2}}(\mu u-v),\;x\in \Gamma_{3D},$$

where $\gamma_{\;ph}=(\kappa {\tilde{L}}_{\;ph}^{2}/D)\eta^{2}$ and the subscript 3*D* indicates the extension of the domains along the *x*_1_ direction between 0 and *L*_*ph*_. On all other boundaries, no-flux boundary conditions are present. We expand *u* and *v* in powers of *η*^2^. Up to *O*(1), this results in

4.3a $$\frac{\partial u_{0}}{\partial t}=\frac{\partial^{2}u_{0}}{\partial x_{1}^{2}}+\frac{1}{\eta^{2}}\frac{\partial^{2}u_{0}}{\partial x_{2}^{2}}+\frac{1}{\eta^{2}}\frac{\partial^{2}u_{0}}{\partial x_{3}^{2}}+\frac{\partial^{2}u_{2}}{\partial x_{2}^{2}}+\frac{\partial^{2}u_{2}}{\partial x_{3}^{2}},\;x\in D_{3D},$$

4.3b $$-n_{1}\frac{\partial u_{0}}{\partial x_{1}}=0,\;x\in \Gamma_{3D},$$

4.3c $$-n_{2}\frac{\partial u_{0}}{\partial x_{2}}-n_{3}\frac{\partial u_{0}}{\partial x_{3}}=\gamma_{\;ph}(\mu u_{0}-v_{0}),\;x\in \Gamma_{3D},$$

4.3d $$\mathrm{and}\;\;\frac{\partial v_{0}}{\partial t}=\frac{\gamma_{\;ph}}{\eta^{2}}(\mu u_{0}-v_{0})+\gamma_{\;ph}(\mu u_{2}-v_{2}),\;x\in \Gamma_{3D}.$$

At the lowest order, the equations are

4.4a $$\frac{\partial^{2}u_{0}}{\partial x_{2}^{2}}+\frac{\partial^{2}u_{0}}{\partial x_{3}^{2}}=0,\;x\in D_{3D},$$

4.4b $$-n_{2}\frac{\partial u_{0}}{\partial x_{2}}-n_{3}\frac{\partial u_{0}}{\partial x_{3}}=\gamma_{\;ph}(\mu u_{0}-v_{0}),\;x\in \Gamma_{3D}$$

4.4c $$\mathrm{and}\;\;\gamma_{\;ph}(\mu u_{0}-v_{0})=0,\;x\in \Gamma_{3D},$$

which leads to the conclusion that *μu*_0_(*x*_1_, *t*) = *v*_0_(*x*_1_, *t*). At the next order,

4.5a $$0=\frac{\partial^{2}u_{0}}{\partial x_{1}^{2}}-\frac{\partial u_{0}}{\partial t}+\frac{\partial^{2}u_{2}}{\partial x_{2}^{2}}+\frac{\partial^{2}u_{2}}{\partial x_{3}^{2}},\;x\in D_{3D},$$

4.5b $$-n_{1}\frac{\partial u_{0}}{\partial x_{1}}=0,\;x\in \Gamma_{3D},$$

4.5c $$-n_{2}\frac{\partial u_{2}}{\partial x_{2}}-n_{3}\frac{\partial u_{2}}{\partial x_{3}}=\gamma_{\;ph}(\mu u_{2}-v_{2}),\;x\in \Gamma_{3D}$$

4.5d $$\mathrm{and}\;\;\frac{\partial v_{0}}{\partial t}=\gamma_{\;ph}(\mu u_{2}-v_{2}),\;x\in \Gamma_{3D}.$$

Integrating over the unit cell and using Gauss’ Law yields

4.6 $$\int_{C}\frac{\partial u_{0}}{\partial t}d^{3}x=\int_{C}\frac{\partial^{2}u_{0}}{\partial x_{1}^{2}}d^{3}x+\int_{\Gamma }\frac{\partial v_{0}}{\partial t}d^{2}x,$$

which, using the relation *μu*_0_ = *v*_0_, yields the following equation for diffusion along the phages:

4.7 $$(|C|+\mu |\Gamma |)\frac{\partial u_{0}}{\partial t}=|C|\frac{\partial^{2}u_{0}}{\partial x_{1}^{2}}.$$

Comparison to equation (3.11) shows that the adsorption factor is equal in all directions of diffusion.

## 5. Results and discussion

The homogenization approach presented here is important for elucidating the nature of the effect of the phages on antibiotic diffusion and the dependence of the model on its key parameters. By writing equation (3.16) in the form of Fick’s law,

5.1 $$\frac{\partial \tilde{u}(\tilde{x},\tilde{t})}{\partial \tilde{t}}=\tilde{\nabla }\cdot \left(\frac{{\tilde{D}}^{\left(\mathrm{eff}\right)}}{\frac{1}{{\tilde{a}}^{2}}(|\tilde{C}|+\tilde{\alpha }|\tilde{\Gamma |})}\tilde{\nabla }\right)\tilde{u}(\tilde{x},\tilde{t}),\;\tilde{x}\in {\tilde{D}}_{H},$$

the effective diffusion time is found to be

5.2 $${\tilde{\tau }}_{D}^{\left(\mathrm{eff}\right)}=\frac{{\tilde{L}}^{2}/{\tilde{a}}^{2}(|\tilde{C}|+\tilde{\alpha }|\tilde{\Gamma |})}{{\tilde{D}}^{\left(\mathrm{eff}\right)}}\equiv \frac{{\tilde{L}}^{2}}{\hat{D}}.$$

Since, without the presence of phages, the diffusion time would be given by ${\tilde{L}}^{2}/\tilde{D}$, by comparison with equation (5.2) we can assess the effect of the phages on the diffusion. More specifically, the relative magnitudes of $\tilde{D}$ and ${\tilde{D}}^{\left(\mathrm{eff}\right)}$ give the strength of the physical phage barrier effect, while the factor $\left(1/{\tilde{a}}^{2})(|\tilde{C}|+\tilde{\alpha }|\tilde{\Gamma |}\right)$ can be used to derive the strength of the adsorption effect.

From equation (5.2), the key parameters governing the model can be derived. In the first place, the effective diffusion time ${\tilde{\tau }}_{D}^{\left(\mathrm{eff}\right)}$ and equation (3.16) do not depend on the adsorption rate $\tilde{\kappa }$. This is expected, since in the derivation of the homogenized model it was assumed that the adsorption time ${\tilde{\tau }}_{\kappa }$ and microscopic diffusion time ${\tilde{\tau }}_{D}$ are of the same order of magnitude. This scale is two orders of ϵ smaller than the macroscopic diffusion time ${\tilde{\tau }}_{D}^{\left(M\right)}$. Hence, compared with ${\tilde{\tau }}_{D}^{\left(M\right)}$, ${\tilde{\tau }}_{\kappa }$ should be small and $\tilde{\kappa }$ should be large. This is equivalent to a high, effectively instantaneous adsorption rate compared with the macroscopic rate of diffusion, and any variation in adsorption rate has no significant effect. Secondly, equation (5.2) indicates a quadratic relationship between the diffusion time and the tactoid width $\tilde{L}$. The dependence of ${\tilde{\tau }}_{D}^{\left(\mathrm{eff}\right)}$ on the equilibrium binding constant $\tilde{\alpha }$ is derived by expanding $\hat{D}$ in powers of $1/\tilde{\alpha }$,

5.3 $$\hat{D}\equiv \frac{{\tilde{D}}^{\left(\mathrm{eff}\right)}{\tilde{a}}^{2}}{\left(|\tilde{C}|+\tilde{\alpha }|\tilde{\Gamma |}\right)}=\frac{{\tilde{D}}^{\left(\mathrm{eff}\right)}}{\tilde{\alpha }|C|((1/\tilde{\alpha })+|\tilde{\Gamma |}/\tilde{\left|C\right|})}\approx \frac{{\tilde{D}}^{\left(\mathrm{eff}\right)}}{\tilde{\alpha }|C||\tilde{\Gamma |}/\tilde{\left|C\right|}}\;⟹\;{\tilde{\tau }}_{D}^{\left(\mathrm{eff}\right)}=\frac{{\tilde{L}}^{2}\tilde{\alpha }|C||\tilde{\Gamma |}/\tilde{\left|C\right|}}{{\tilde{D}}^{\left(\mathrm{eff}\right)}}.$$

Hence, the diffusion time depends linearly on $\tilde{\alpha }$, if $\tilde{\alpha }$ is sufficiently large.

**Figure 3.**
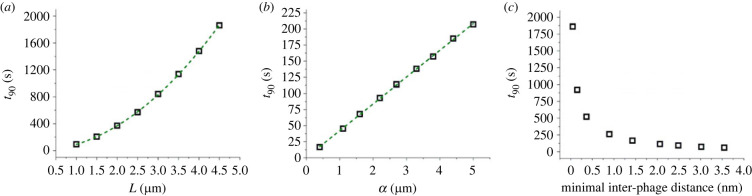
Dependence of the equilibration time on (*a*) the tactoid width, with the dashed line showing a quadratic fit, (*b*) the binding equilibrium constant α, with a linear fit indicated by the dashed line, and (*c*) the phage packing density.

The parameter dependence of the model was verified by integrating the microscopic equations in Comsol. As expected from the previous parameter analysis, for fast adsorption (${\tilde{\tau }}_{\kappa }\ll {\tilde{\tau }}_{D}^{\left(M\right)})$, the value of $\tilde{\kappa }$ has no influence on the results. Figure 3 shows the dependence of the numerically calculated diffusion time on various parameters. The diffusion time is quantified as *t*_90_, the time at which the antibiotic concentration that has reached the bacterium is 90% of the equilibrium concentration. To verify that the results are no artefact at *t*_90_, calculations were repeated for *t*_10_, the time at which the concentration at the bacterium is 10% of the equilibrium (see figure S1 in the electronic supplementary material). The results demonstrate a linear dependence of the diffusion time on $\tilde{\alpha }$ and a quadratic dependence on $\tilde{L}$, as discussed above. Furthermore, the diffusion time increases strongly with increasing packing density, quantified as the minimal distance between phages. This is expected, since as this distance approaches zero, there is no space left between phages for the antibiotics to diffuse through and the diffusion time diverges.

As demonstrated here, homogenization allows us to find an expression for the diffusion time and to assess its dependence on the key model parameters, without the need to solve the microscopic model numerically. The biological implications of this are that the mechanism of liquid-crystal-induced antibiotic tolerance can be analysed more efficiently and with more insight into the parameter significance than numerical analysis can provide. More specifically, the formation of liquid crystals by Pf4 phages has been shown to increase antibiotic tolerance; the model outlined in this paper elucidates the importance of diffusion barrier and adsorption effects to this increased tolerance. This way, we can clarify whether these are the principal effects behind the increased antibiotic tolerance, or if other mechanisms are more important. A more in-depth analysis of this application is reported elsewhere [25].

**Figure 4.**
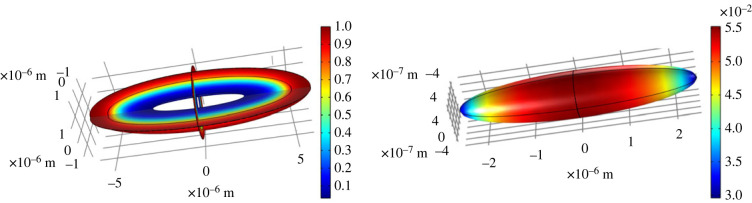
Homogenized modelling of an entire tactoid at *t* = 100 s, three slices of which are shown on the left. The colour scale indicates antibiotic concentration. The image on the right shows the antibiotic concentration on the bacterium surface. The concentration of the phages was varied along the long axis of the tactoid, in such a way that the diffusion coefficient varies as shown in figure 5.

**Figure 5.**
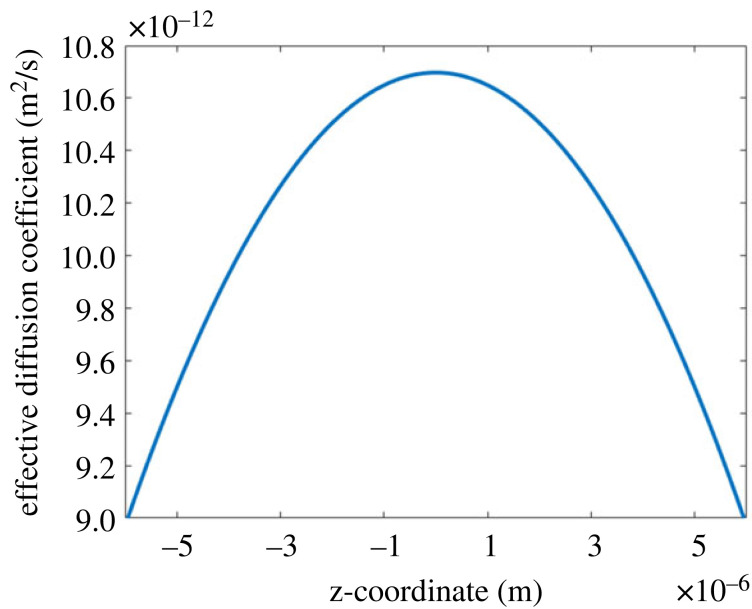
The effective diffusion coefficient along the tactoid length.

To assess the influence of varying phage packing density on the antibiotic diffusion, we also model a tactoid in three dimensions. The results are shown in figure 4. The boundary conditions are the same as for the two-dimensional model, as is the addition of a small outer layer around the tactoid. The packing of the phages was taken to be twice as dense at the tips of the tactoid as in the middle, since simulations have shown denser packing at these locations [27]. The packing density across the tactoid was varied in such a way that the effective diffusion coefficient follows the quadratic trend shown in figure 5. The diffusion coefficient is anisotropic, as discussed in §4. It is governed by the orientation of the phages; since these align along the ellipsoidal bacterium, their orientation is given by the equation for this ellipsoid. Figure 4 shows that the variation in packing density has little influence on the diffusion time. This can be explained by the relatively fast diffusion along the phages, which homogenizes the antibiotic concentration. Furthermore, the influence of packing density on diffusion time is only strong at the highest phage concentrations (figure 3), which are beyond the concentrations modelled here.

**Figure 6.**
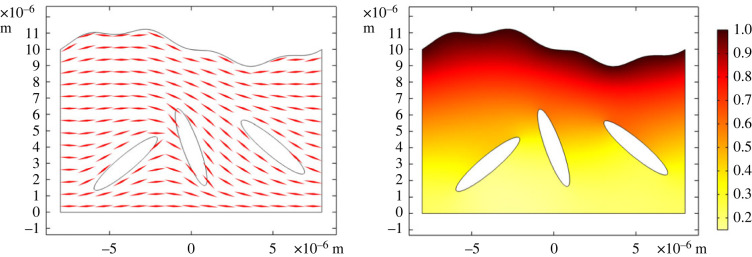
The modelling of antibiotic diffusion in a layer of Pf4 liquid crystal of irregular thickness with embedded bacteria (shown as ellipsoids) at *t* = 1000 s. The left image shows the orientation of the phages, indicated by red lines. On the right, the antibiotic concentration is indicated by the colours.

*In vivo*, tactoids have not been observed directly in liquid crystalline *P. aeruginosa* biofilms; however, the biofilm contains high concentrations of similar depleting polymers, with a liquid crystalline structure forming on the global biofilm scale [4]. Therefore, the application of our model to tactoids, corresponding to *in vitro* systems, is a first step towards modelling a biofilm in its full complexity. Furthermore, our results concerning increased antibiotic tolerance apply to embedding of bacteria in a liquid crystalline environment of any form and are therefore not limited to *in vitro* systems. To illustrate this, the homogenized model was finally used to analyse antibiotic diffusion in a layer of Pf4 liquid crystal of varying thickness with embedded bacteria. The geometry of the latter model corresponds more closely to an *in vivo* biofilm than a tactoid. The results are shown in figure 6. In this liquid crystalline layer, antibiotics are continually added from the upper boundary, using the same Dirichlet boundary condition as for the edge of the outer domain in the tactoid model. There is no flux of antibiotics at the lower boundary. The profile of the upper boundary was modelled using sinusoidal functions to ensure horizontal periodicity, with the equation *y* = 10 + (1/7)sin ((*x* + 8)*π*/2) + (1/3)sin ^2^((*x* + 8)*π*/4) + sin ((*x* + 8)*π*/8) with *x* and *y* in μm. The orientation *θ* of the phages is solved by the equation $\nabla^{2}\theta =0$ (the Frank–Oseen model in the one electric constant approximation without external fields), with planar alignment at the phages and upper and lower boundaries. This orientation, which determines the diffusion tensor, is indicated in figure 6 by red lines. The antibiotics reach the bacteria on longer timescales than in a tactoid, which is expected given the influence of the liquid crystalline layer thickness on the diffusion time shown in figure 3. Of note is that diffusion is slower in the narrower space between the two leftmost bacteria, as expected. The results of these models show the flexibility of homogenization for the modelling of both *in vitro* and *in vivo* systems, for various geometries, and in both two and three dimensions. In other words, the homogenized equations derived in this paper are applicable not only to the specific biological systems considered here, but also to similar but more complex biological systems and general diffusion in porous media.

In view of this general applicability, certain restrictions and assumptions that homogenization entails, besides local periodicity, need to be addressed. The results of homogenization depend on the scaling choices of the model parameters. Most of the scaling choices made in this paper follow straightforwardly from realistic requirements. For example, the scaling of $\tilde{\alpha }$, and the relative scales of $\tilde{u}$ and $\tilde{v}$, are determined by requiring that the total amounts of free and bound antibiotics are of the same order and do not diverge. An exception is the relative scaling of diffusion and adsorption times; this means adsorption is much faster than macroscopic diffusion, which is reasonable, but other scalings could also be possible. A benefit of the scaling choice adopted is that it gives the most general, nontrivial results; for instance, if the adsorption rate were slower, adsorption would be negligible on a microscopic scale. Then *u* and *v* decouple at this scale, and are determined by a separate set of equations. For an even slower adsorption rate, adsorption would be completely negligible in the homogenized equations. For an even faster adsorption rate, the homogenized model is identical to the model presented in this paper, since as long as adsorptive equilibrium is reached long before diffusive equilibrium, it does not matter how fast adsorption is from a macroscopic point of view. The different scaling choices are summarized in table 1. For an overview of the applicability of homogenization for a wide array of scaling choices, applied to the closely related topic of reactive diffusion in porous media, we refer to the work of Battiato & Tartakovsky [13].

**Table 1.** Summary of scaling regimes of the diffusion and adsorption times, and the resulting homogenized models.

scaling	description of homogenized model
${\tilde{\tau }}_{\kappa }\ll {\tilde{\tau }}_{D}\ll {\tilde{\tau }}_{D}^{\left(M\right)}$	The same homogenized model as presented in this paper.
${\tilde{\tau }}_{D}∼{\tilde{\tau }}_{\kappa }\ll {\tilde{\tau }}_{D}^{\left(M\right)}$	The scaling presented in this paper: an effective diffusion equation with a time coefficient determined by adsorption.
${\tilde{\tau }}_{D}\ll {\tilde{\tau }}_{\kappa }∼{\tilde{\tau }}_{D}^{\left(M\right)}$	Adsorption and diffusion decouple at the microscopic scale.
	Homogenization yields two coupled macroscopic equations: an effective diffusion and a trivial adsorption equation.
${\tilde{\tau }}_{D}\ll {\tilde{\tau }}_{D}^{\left(M\right)}\ll {\tilde{\tau }}_{\kappa }$	A homogenized diffusion equation without adsorption: slow global, uniform adsorption follows diffusive equilibrium.

## 6. Conclusion

The results presented in this work demonstrate that homogenization can be applied to describe and quantify the diffusion and adsorption of antibiotics in liquid crystals consisting of filamentous phages. We identify that adsorption is a key ingredient in increasing the antibiotic diffusion time and that it may therefore significantly enhance antibiotic tolerance. The advantage of homogenization is that it allows us to link the diffusion time to biological parameters. This is a preliminary study of liquid crystals in biology, which was chosen for its simplicity and because the system is well-studied and well-controlled. Since the applicability of homogenization only depends on local structural regularity, it can be applied to more complex structures such as biofilms and other biological systems with complex geometries that change slowly over a microscopic length scale. This yields analytically solvable models, the physical and chemical variables of which can be linked to the biological properties of the system.

## Data Availability

The data are provided in electronic supplementary material [28].
